# Differential Effects of Leptin and Adiponectin in Endothelial Angiogenesis

**DOI:** 10.1155/2015/648239

**Published:** 2015-01-15

**Authors:** Raghu Adya, Bee K. Tan, Harpal S. Randeva

**Affiliations:** ^1^Division of Translational and Systems Medicine-Metabolic and Vascular Health, Warwick Medical School, University of Warwick, Coventry CV4 7AL, UK; ^2^Department of Obstetrics and Gynaecology, Birmingham Heartlands Hospital, Birmingham B9 5SS, UK

## Abstract

Obesity is a major health burden with an increased risk of cardiovascular morbidity and mortality. Endothelial dysfunction is pivotal to the development of cardiovascular disease (CVD). In relation to this, adipose tissue secreted factors termed “adipokines” have been reported to modulate endothelial dysfunction. In this review, we focus on two of the most abundant circulating adipokines, that is, leptin and adiponectin, in the development of endothelial dysfunction. Leptin has been documented to influence a multitude of organ systems, that is, central nervous system (appetite regulation, satiety factor) and cardiovascular system (endothelial dysfunction leading to atherosclerosis). Adiponectin, circulating at a much higher concentration, exists in different molecular weight forms, essentially made up of the collagenous fraction and a globular domain, the latter being investigated minimally for its involvement in proinflammatory processes including activation of NF-*κβ* and endothelial adhesion molecules. The opposing actions of the two forms of adiponectin in endothelial cells have been recently demonstrated. Additionally, a local and systemic change to multimeric forms of adiponectin has gained importance. Thus detailed investigations on the potential interplay between these adipokines would likely result in better understanding of the missing links connecting CVD, adipokines, and obesity.

## 1. Introduction

Obesity is a global epidemic with serious health complications. In particular, obesity is associated with elevated free fatty acid levels, leading to the development of insulin resistance, diabetes, and cardiovascular disease (CVD) [1–3]. The development of CVD is characterised by impaired nitric oxide (NO) release from vascular endothelium and decreased blood flow to insulin target tissues resulting in insulin resistance, termed as endothelial dysfunction [4]. However, the mechanisms by which obesity causes both insulin resistance and vascular dysfunction are not fully understood. In this respect, increasing attention has been paid to the direct vascular effects of adipose tissue (AT) derived factors termed “adipokines” or “adipocytokines” which have been documented to affect endothelial function [5]. A few of these adipokines are characterised by their favourable effects to maintain the body's energy and vascular homeostasis; however, adipokines have also been implicated in the pathogenesis of obesity-related disorders, such as atherosclerosis, specifically, by increasing the expression of proangiogenic/proatherogenic factors like endothelial gelatinases (matrix metalloproteinases 2 and 9) and vascular endothelial growth factor (VEGF) [6]. Leptin, adiponectin, monocyte chemoattractant protein- (MCP-) 1, plasminogen activator inhibitor- (PAI-) 1, tumour necrosis factor (TNF) *α*, interleukin- (IL-) 6, and resistin are a few of these adipokines implicated in endothelial dysfunction [5].

## 2. Obesity and Molecular Aspects of Endothelial Dysfunction

Endothelial dysfunction in obesity is characterised by increased generation of oxygen-derived free radicals (ROS) [7]. This is contributed by vascular cells and inflamed hypertrophied adipocytes as a result of endoplasmic reticulum (ER) stress and mitochondrial dysfunction [8]. Enzymes of mitochondrial electron transport chain, xanthine oxidase, cyclooxygenases, lipoxygenases, myeloperoxidases, cytochrome P450 monooxygenase, heme oxygenases, peroxidases, and NAD(P)H oxidases contribute to endothelial dysfunction [7]. Uncoupling of endothelial nitric oxide synthase (eNOS) is a major contributor to ROS production [9]. This results in decreased NO (nitric oxide) bioavailability, increased O_2_^−^ production, and formation of peroxynitrite (ONOO^−^), a key mediator of lipid peroxidation and foam cell formation in atherosclerotic lesions [10]. Additionally, ROS accumulation results in activation of signalling cascades that regulate transcription factors, including NF-*κβ* (nuclear factor kappa beta) adhesion molecules, chemotactic factors, antioxidant enzymes, and vasoactive substances promoting adhesion and migration of circulating monocytes initiating atherosclerotic lesions [11]. Dysregulated adipokine production leading to increased ROS generation forms a major feedback loop in initiation, maintenance, and progression of endothelial dysfunction [12].

Adiponectin and leptin are the two widely studied most-abundant, circulating adipokines. In this review, we discuss the diverse roles of leptin and adiponectin in endothelial dysfunction with emphasis on proangiogenic/proatherogenic factors in the endothelial cells.

## 3. Adiponectin

Adiponectin, the most abundantly secreted adipokine (2–20 *μ*g/mL in circulating plasma), was first identified as Acrp30—adipose complement-related protein of 30 kDa [13]—because of its high similarity to complement protein. Adiponectin exerts its insulin-sensitizing effects by increasing *β*-oxidation of fatty acids, in the process reducing serum triglyceride and levels of free-fatty acids, and thus indirectly improving insulin sensitivity of the liver [14]. In addition to its metabolic actions, adiponectin is also reported to possess antiatherogenic and anti-inflammatory properties [15]. Circulating low adiponectin levels (hypoadiponectinemia) is considered an independent risk factor for endothelial dysfunction and modulating vessel wall health [16].

Adiponectin is a 247-amino acid protein with four domains, an amino-terminal signal sequence, a variable region, a collagenous domain, and a carboxy-terminal globular domain [13, 17–19], and undergoes posttranslational modifications within the adipocytes into multimeric forms including trimers, hexamers, and high-molecular-weight (HMW) oligomers [20]. More importantly, cleavage of globular domain of full-length adiponectin (fAD) by activated monocytes has been reported to conversely affect the protective role of fAD [21].

**Figure 1:**
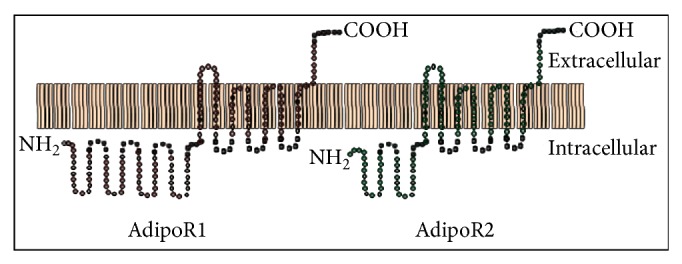
Structure of adiponectin receptors—AdipoR1 and AdipoR2 (66.7% amino acid homology).

Both globular adiponectin (gAD) and fAD exert their effects* via* transmembrane G-protein coupled receptors, adiponectin receptor 1 (AdipoR1), and adiponectin receptor 2 (AdipoR2) [22]. These receptors have been described as structurally related integral plasma membrane proteins with seven transmembrane domains having their extracellular C terminus and intracellular N terminus regions [depicted in Figure 1]. AdipoR1 is abundantly synthesised and expressed in skeletal muscle, whereas AdipoR2 is found predominantly in liver [22]. Both receptors have also been described in pancreatic *β*-cells, macrophages, endothelial cells, and smooth muscle cells within atherosclerotic plaques [23, 24]. C-terminus of AdipoR1 possesses high affinity for gAD, whereas adipoR2 exhibits intermediate affinity for both the gAD and fAD. Overexpression and gene knockout experiments in rodents have demonstrated the ability of these receptors to activate AMP-activated protein kinase (AMPK), p38 mitogen activated protein kinase (p38 MAPK), and peroxisome-proliferator-activated receptor-*α* (PPAR-*α*) and to stimulate fatty acid oxidation and glucose uptake in murine hepatocytes and C2C12 myocytes [22]. Globular, trimeric, and high-molecular-weight (HMW) adiponectin forms activate different signal transduction pathways [25]. Additionally, osmotin, a plant protein with structural similarities to mammalian globular adiponectin, binds to adiponectin receptors and activates AMPK in C2C12 myocytes [26].

Exercise training increases AdipoR1/R2 mRNA expression in human skeletal muscle [27], whereas no significant change has been reported in human subcutaneous adipose tissue during calorie restriction [28]. The expression levels of AdipoR1 and AdipoR2 in skeletal muscle, as well as plasma adiponectin concentrations, have been described to be lower in individuals with a family history of type 2 diabetes mellitus (T2DM) than in those with no family history [29]. The expression level of both receptors correlated positively with insulin sensitivity [29]. A study by Zhang et al., 2010, had demonstrated significant reduction in expression of AdipoR2 in both coronary arterioles and aortas of diabetic mice, with no changes in AdipoR1 expression levels [30].

Another adiponectin-binding protein with a preference for HMW adiponectin multimers and no affinity for the trimeric adiponectin has been identified as T-cadherin [45]. Since this protein is a glycosylphosphatidylinositol-anchored extracellular protein devoid of any intracellular domain, the mechanism explaining its role in adiponectin intracellular signalling has not been fully clarified.

## 4. Leptin

Leptin, a 16 kDa protein, is an adipose-tissue specific adipokine involved in regulation of food intake and energy haemostasis [46]. Leptin also has multiple roles in carbohydrate and lipid metabolism, reproductive system, and inflammatory and immune reactions [47]. Leptin has been shown to exert atherogenic, thrombotic, and angiogenic actions on the vasculature [48–50]. It has been linked extensively with obesity leading to CVDs including atherosclerosis, myocardial infarction, and stroke [51, 52].

**Figure 2:**
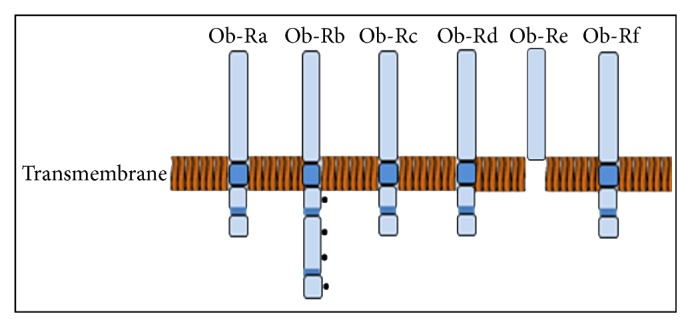
Structure of leptin receptor isoforms—6 different isoforms of the leptin receptor Ob-R (a–f). Extracellular ligand-bind domains of receptor isoforms are identical but they differ at the C-terminus.

Leptin acts on target cells through plasma membrane receptors (Figure 2) and exists in at least six isoforms, Ob-Ra through Ob-Rf, signalling predominantly via JAK/STAT (Janus kinases/signal transducers, and activators of transcription) pathway [53]. Functional leptin receptors (both short and long forms of OB-R) have been identified on endothelial cells [38, 54], and numerous studies link the possible mechanisms responsible for leptin-induced endothelial dysfunction. JAK-2/IRS-2/PI3-K/Akt pathways and nuclear translocation of STAT (signal transducer and activator of transcription) proteins have been implicated to play a pivotal role in leptin-mediated effects in endothelial cells [53, 55]. Interestingly, in states of obesity despite a paradoxical impairment of the satiety response, leptin resistance does not extend to leptin mediated endothelial dysfunction [56–59]. Recent studies have demonstrated increased expression and modulation of astrocytic leptin receptor subtypes (Ob-R) in adult-onset obesity facilitating increased leptin transport via the human brain endothelial cell barrier [60]. Although this selective response of leptin has gained much attention, the molecular basis remains poorly understood.

### 4.1. Adiponectin, Nitric Oxide (NO), and Endothelial Adhesion Molecules

Decreased production of NO by the endothelial cells is considered as the hallmark of endothelial dysfunction. Adiponectin has been reported to increase NO production in endothelial cells by the activation of phosphotidylinositol-3 (PI-3) kinase/Akt signalling pathway [61]. Furthermore, the involvement of AMPK and protein kinase A [PKA, or cyclic AMP- (cAMP-) dependent protein kinase] signalling have also been implicated to play a major role in both adiponectin induced NO production and suppression of endothelial ROS generation, inhibiting endothelial NF-*κβ* (nuclear factor kappa beta) signalling [32, 62–65].

Animal studies conducted in adiponectin knockout (KO) mice have shown a significant reduction in endothelium-dependent vasodilatation [66]. Adiponectin and lectin-like oxidized LDL (ox-LDL) receptor- (LOX-) 1 have been demonstrated to exhibit a reciprocal pattern in states of endothelial dysfunction and inflammatory insults. Adiponectin administration in apolipoprotein E (ApoE) knockout (KO) mice restored NO-mediated endothelium-dependent vasorelaxation and decreased aortic LOX-1 expression, implicating a key biological function of adiponectin in reducing systemic oxidative mediators and ox-LDL uptake [67]. More importantly, studies in T2DM mice have elucidated a similar reciprocal regulation between adiponectin and TNF-*α* affecting the regulation of both coronary and aortic endothelial function. These effects seem to be regulated by a common downstream transcription factor-NF-*κβ* [68]. Additionally,* in vivo* studies have indicated the critical role of adiponectin in alleviating sepsis-induced microvascular dysfunction leading to blood brain barrier (BBB) dysfunction in the mouse brain* via* modulation of E-selectin expression [69].

Clinical studies have demonstrated impaired production of eNOS in the vasculature consequently leading to decreased endothelium-dependent vasorelaxation in subjects with decreased adiponectin levels. Furthermore, adiponectin drastically improves oxidized LDL induced decrease in eNOS activity [36, 70]. More importantly, to simulate pathological states of obesity and diabetes, the role of adiponectin in hyperglycaemic/hyperinsulinaemic environments has been studied. Xiao et al. have demonstrated a protective action of gAd in alleviating endothelial dysfunction caused due to intermittent hyperglycaemia, implicating the involvement of Akt, AMPK, and eNOS signalling pathways [71]. The deleterious effects of hyperglycaemia in obese and diabetic subjects extend to a concomitant decrease in circulating endothelial progenitor cells (EPCs) leading to impaired endothelial repair. Studies have indicated that gAd promoted EPC migration and tube formation and dose-dependently upregulated phosphorylation of eNOS, Akt, and augmented NO production. Additionally,* in vivo* results have revealed that gAd rescued high glucose induced impairment of EPC functions by restoration of eNOS activity and vasculogenesis [37].

It is interesting to note that adiponectin induced activation of eNOS as well as increased production of NO by the endothelial cells is crucial in mediating its anti-inflammatory effects. In this context,* in vivo* studies have demonstrated that pharmacological blockade of eNOS leads to decreased protective effect imparted by adiponectin, leading to increased leukocyte adhesion by TNF-*α* [72].

Endothelial dysfunction includes the activation of endothelial adhesion molecular cascade critical in facilitating the entry of macrophages into the vessel wall [73]. Circulating low adiponectin levels in metabolic diseases like obesity and diabetes have been linked to the triggering of an inflammatory signalling cascade, leading to the early development of atherosclerosis [74]. The development of a similar scenario in* Adipoq–/–* (adiponectin knock-out mice) mice further strengthens the association between adiponectin and CVD [72]. Adiponectin replacement therapy reversed the microvascular inflammatory changes in these* Adipoq–/–* mice. Furthermore, adiponectin has been shown to inhibit the vascular inflammatory response of endothelium to TNF-*α* induced activation of NF-*κβ* and increased expression of adhesion molecules vascular cell adhesion molecule (VCAM-1), intercellular adhesion molecule (ICAM-1), and endothelial selectin (E-selectin) [32]. Functional effects induced by mediators of systemic inflammation like TNF-*α* and subsequent interactions with adipokines have a significant influence in either promoting or downregulating vascular insult.

### 4.2. Adiponectin and Endothelial Angiogenesis

Adiponectin has been shown to induce* in vitro* angiogenesis in endothelial cells* via* AMPK-eNOS pathway [31]. More importantly, adiponectin replacement rectified ischemic stress induced impaired angiogenesis in* Adipoq–/–* mice [75]. Studies conducted in adiponectin-overexpressed mice brain (following transfection with adenoassociated viral vector (AAV) containing adiponectin gene) have shown a significant benefit induced by adiponectin following ischemic insult. This protective action was related to adiponectin induced focal angiogenesis involving VEGF and AMPK pathways [76]. On the other hand, other groups have demonstrated the potent inhibition of endothelial angiogenic events like migration and proliferation by adiponectin [33], involving MAPK and cAMP-PKA pathways [34]. Similar antiangiogenic effects of adiponectin have been studied in tumour growth suppression involving Rho kinase/IFN-inducible protein 10 and matrix metalloproteinase 9 (MMP-9) [77].

### 4.3. Differential Effects of fAD and gAD

Numerous studies have implicated the vasoprotective actions of fAD by reducing the expression of endothelial adhesion molecules and inhibiting TNF-*α* induced cytokine production from macrophages* via* NF-*κβ*/cAMP-dependent pathway [32, 78–80]. Animal studies in Ad^−/−^ (mice completely lacking adiponectin) and Ad^+/−^ (adiponectin-hemizygous mice) mice showed an increased expression of E-selectins and VCAMs. Moreover, administration of gAd attenuated VCAM expression in Ad^−/−^ mice [72]. Adiponectin has been demonstrated to suppress VEGF-stimulated HCAEC migration* via* cAMP/PKA-dependent signalling [34]. Clinical studies in patients with acute coronary syndromes have shown a negative correlation between circulating adiponectin levels and MMP-9/TIMP-1 ratio, an independent predictor of atherosclerotic plaque stability [80]. However, three independent studies have demonstrated that gAd activates NF-*κβ* leading to activation of the proinflammatory adhesion cascade, proliferation, and increased procoagulability in endothelial cells and cardiac fibroblasts [39, 40, 81].

Furthermore, studies by Hattori et al. have indicated the suppression of cytokine induced inflammatory cascade* via* NF-*κβ* by gAD, albeit with a prolonged response time. The authors attribute this to desensitisation of the receptor, seen in instances of cytokine overload. More recently, colocalization studies conducted by Xu et al. have demonstrated that adiponectin induces interaction between lymphotoxin- (LT-) b receptor (LTBR) and human AdipoR1, subsequently resulting in inhibition of the NF-*κβ* pathway [82].

Studies have demonstrated the ability of leukocyte elastase secreted by activated monocytes and neutrophils to cleave the globular domain of adiponectin [21]. This local generation of gAd at sites of inflammation, namely, in atherosclerotic lesions, could be having pathophysiological relevance given the differential actions of multimeric forms of adiponectin. In a study comparing the differential effects of fAd and gAd in human aortic endothelial cells (HAEC), both peptides upregulated NO production by AMPK-dependent pathways. However, in contrast to fAd, gAd activated NF-*κβ* and p38 MAPK signalling pathways, resulting in cyclooxygenase-2 (COX-2) production and subsequently prostacyclin 2 [PGI] release. This study further demonstrated that monocyte-endothelial adhesion enhanced by gAD remained unaffected with either abrogation of AdipoR1 [siRNA] signalling or COX-2 [siRNA] downregulation, thereby suggesting independent mechanisms governing actions of fAd and gAd [41].

The obvious discrepancies between the experimental outcomes could be due to the differences in the forms of adiponectin used (Table 1). Additionally, endogenous production of adiponectin by the endothelial cells needs to be considered [83].

**Figure 3:**
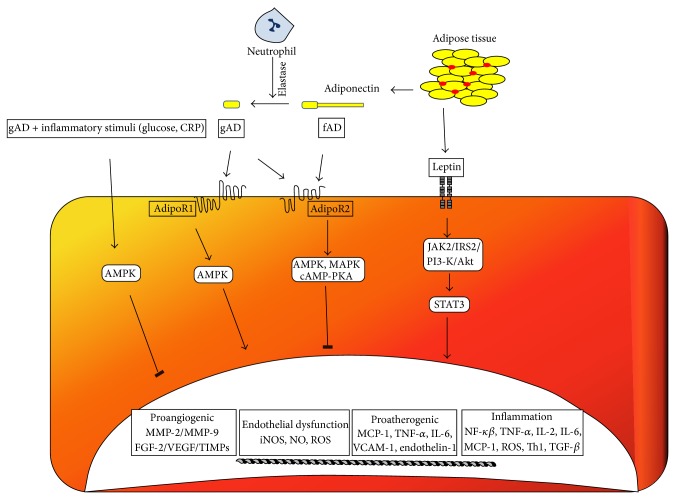
Differential effects of leptin and adiponectin in vascular endothelium. Dual effects of gAD and fAD on endothelium with and without inflammatory stimuli. Circulating fAD gets cleaved by leucocyte elastase (secreted from neutrophils) releasing globular domain (gAD) fraction. AdipoR1 and AdipoR2 receptors following engagement with fAD, signals downstream activating the following pathways (a) AMPK, (b) cAMP-PKA, (c) MAPK, and (d) PI3K-Akt. Activation of cAMP-PKA/AMPK causes increased NO production, decreased ROS generation, suppression of NF-*κβ* pathway leading to reduction in IL-18, and endothelial adhesion molecule expression. These events collectively lead to a decrease in EC permeability, motility, and migration. Activation of AMPK/PI-3k/Akt signalling pathway specifically leads to eNOS phosphorylation and NO release.* In vitro* studies have shown that gAD independently activates NF-*κβ* via AdipoR1/AMPK-Akt pathway. Proangiogenic/inflammatory effects of gAD have been shown to involve AMPK-Akt pathways. However, these pathways (AMPK-Akt) also contribute to an opposite effect of gAD in coexisting states of hyperglycaemia and inflammation. In hyperglycaemic and hyperinsulinaemic states, gAD improves endothelial dysfunction* via* activation of Akt-AMP-eNOS pathways and suppression of endothelial ROS generation* via* inhibition of NF-*κβ* signalling. The binding of leptin to its receptor (OB-Rb) leads to the phosphorylation of Ob-R/JAK2 complex. Subsequent activation of downstream signalling cascades including PI3k/Akt-STAT3 activation results in transcription of genes [MCP-1, TNF-*α*, IL-6/-2, and endothelin-1] involved in proatherogenic/angiogenic and inflammatory effects, potentiating endothelial proliferation. Additionally, leptin signalling in ECs also activates endothelial cell adhesion molecules, MMPs, and VEGF resulting in impaired endothelium-dependent vasodilatation promoting hypertension and atherosclerosis.

Recently, we undertook a study to investigate the effect of gAD and fAD (Figure 3) on endothelial cell proliferation as well as* in vitro* migration and angiogenesis in relation to the induction of endothelial angiogenic factors, specifically, MMP-2, MMP-9, and VEGF; furthermore, we examined the involvement of the adiponectin receptors, that is, adiponectin receptor 1 (AdipoR1) and adiponectin receptor 2 (AdipoR2), within this context [35]. More importantly, given the connection between the coexistence of hyperglycaemia and systemic inflammation with vascular disease in pathological states such as diabetes mellitus, we also studied the interaction between glucose and C-reactive protein (CRP) [a potent proinflammatory protein], respectively, with gAD and fAD. Finally, since AMP-activated protein kinase (AMPK), a stress-activated protein kinase, and Akt have been implicated as critical mediators of adiponectin induced angiogenesis in both normoxic and ischemic tissues, we examined the role of these signalling pathways in gAd induced endothelial angiogenesis [31]. We found that gAd led to a significant increase in* in vitro* endothelial proliferation, migration, and angiogenesis with concomitant increase in MMP-2, MMP-9, and VEGF gene and protein production, as well as MMP-2 and MMP-9 activation. The effect of gAd on VEGF appears to be mediated by AdipoR1 whereas the effect of gAd on MMP-2 and MMP-9 appears to be mediated by AdipoR1 and AdipoR2. On the other hand, only endothelial cell proliferation was significantly increased by fAd and appears to be mediated by AdipoR2; no significant effects on MMP-2, MMP-9, and VEGF were observed. Ouchi et al. 2004 had reported that mouse fAD stimulates* in vitro* migration and angiogenesis and suggested that this effect may be beneficial in line with the report by Shibata et al., 2004, who demonstrated that adiponectin promotes ischemia-mediated revascularization in adiponectin-knockout mice. It is important to note that although* in vitro* angiogenic assays have been merited as useful reporters in deciphering specific steps, they however lack the complex interplay of multiple factors vital for* in vivo* processes [31, 33]. Taken together, it remains unclear as to whether our observations reflect on balance a beneficial or detrimental effect of adiponectin.

Thus it seems imperative to study the local effects of various multimers of adiponectin* in situ*, for instance, in atherosclerotic plaques, to ascertain the potential pro/anti-inflammatory actions of this adipokine.

### 4.4. Leptin and Endothelial Cell Dysfunction

Leptin has multiple proinflammatory and immune mediated effects on the vasculature. On engagement with leptin receptors expressed on vascular cell walls, leptin induces oxidative stress responses, increases MCP-1, TNF-*α*, IL-6, and endothelin-1, and potentiates proliferation, along with the expression of other endothelial cell adhesion molecules, MMPs, VEGF, and impaired smooth-muscle cell function, resulting in impaired endothelium-dependent vasodilatation promoting hypertension and atherosclerosis [84]. Clinical studies have reported a positive correlation between circulating leptin, plasma thrombomodulin, and VCAM-1 levels [85].

### 4.5. Leptin Induced Endothelium Dependent and Independent Vasodilation

Endothelium dependent leptin induced vasorelaxation observed in rat arterial rings was promptly inhibited by increasing extracellular calcium [86] and inhibition of NO synthase. Moreover, leptin has been demonstrated to phosphorylate eNOS leading to NO release [44]. Intra-arterial administration of leptin showed a similar vasoactive response independent of NO in humans [87]. Additionally, a direct vasorelaxive effect of leptin on smooth muscle cells independent of endothelium was also observed in both rat and human arterial samples [88, 89]. Acute hyperleptinemia induced vasodilatory effects and this seemingly contradicts the coexisting hypertension and increased leptin levels on obesity. A plausible explanation for this could be attributed to the acute and chronic effects of leptin on the vasculature. Recent* in vivo* studies have revealed additional induction of endothelial nNOS (neuronal nitric oxide synthase) expression by leptin as a compensatory mechanism to induce endothelium-dependent relaxation in eNOS (−/−) mice [90]. More importantly, hyperleptinemia induced endothelial dysfunction may play a crucial role in the differential actions of leptin.

### 4.6. Leptin Induced Endothelial Dysfunction and NO Production

Experiments by Naseem have indicated that leptin initiated upregulation of inducible NO synthase (iNOS), which may or may not lead to net increased NO production and paradoxically impairs endothelial function by inducing oxidative stress [91]. Furthermore, a significant vasodilatory response induced by leptin in lean Zucker rats failed to do so in obese hyperleptinemic Zucker rats [92].

As mentioned, leptin has been shown to induce oxidative stress by increasing the formation of reactive oxygen species (ROS), a key mediator of endothelial dysfunction [42, 93]. This generated ROS has potent peroxidant effects and thereby reduces the bioavailability of NO in aortic endothelial cells [93], vascular smooth muscle cells [84], and macrophages [94]. Additionally, ROS further contributes to endothelial dysfunction by upregulating proinflammatory cascades including adhesion and chemotactic pathways in endothelial cells [95].

It is interesting to note that genetically modified (ob/ob- leptin knock out) mice maintain a relative hypotensive status in comparison with their wild types. Leptin administration in these mice promptly induces hypertension. This could be attributed to the disturbance in the fine balance between the sympathetic nervous system and endothelial cell mediated regulation of vasomotor tone [96]. With respect to the regulation of leptin receptors and endothelial dysfunction, a study by Park et al. 2012 has revealed that leptin receptors in coronary arterioles are downregulated in high-fat fed sedentary mice leading to endothelial dysfunction. However, when subjected to exercise, the expression of leptin receptors in coronary arterioles was restored along with maintenance of eNOS phosphorylation, leptin sensitivity, and redox balance [97].

### 4.7. Leptin and Endothelial Angiogenesis

As mentioned previously, leptin-mediated actions in endothelial cells, including angiogenesis, primarily occur* via* the activation of Ob-R. It is interesting to note the increased expressions of both Ob-R and MMPs in atherosclerotic plaques, particularly the endothelial lining of neointimal regions, suggesting the role of leptin in mediating aberrant angiogenesis [98]. Both* in vivo* and* in vitro* studies have demonstrated the activation of endothelial Ob-R by leptin, leading to capillary tube formation, a prerequisite for angiogenesis [38]. Bouloumié et al. showed that leptin induced activation of mitogen-activated protein kinase family ERK1/2 leads to an increase in endothelial cell viability in serum-free media. Leptin has been shown to upregulate key proangiogenic molecules like the gelatinases (MMPs, MMP-2/-9) and TIMPs. Additionally, leptin has been shown to upregulate and act synergistically with the key angiogenic mediators like FGF-2, VEGF, and its receptor VEGFR1, stimulating vascular permeability, consequently resulting in functional angiogenesis [99].

It is important to note that wound healing disorder (due to deficient angiogenesis) in ob/ob mice is corrected by both topical and systemic leptin administration but not in* fa/fa* Zucker rats (rats with a recessive trait of the leptin receptor), due to the absence of functional leptin receptors [100]. In a recent study involving an obese NZO (mice with phosphatidylcholine transfer protein mutation leading to abnormal lipid homeostasis) mice model, the angiogenic potential of leptin was found to be insignificant, perhaps due to the relative inactivity of its receptor in these mice [101]. Studies in HUVECs have implicated the involvement of a functional endothelial p38 (MAPK)/Akt/COX-2 signalling axis for leptin's proangiogenic effects and more importantly this signalling pathway is regulated upstream by ObRb-dependent activation of VEGFR2 receptor [102].* In vivo* findings have implicated increased mobilisation of vascular progenitor cells mobilized from the bone marrow in response to leptin stimulation leading to angiogenesis. These effects of leptin seem to be mediated* via* Ob-R induced activation of NOX2 and MMP9 [103]. Additional studies have evidenced the importance of an ObR-Src kinase-alpha v beta 5 cross talk in leptin mediated functional effects in enhancing the angiogenic potential of circulating angiogenic cells (CACs). More importantly, CACs derived from obese, hyperleptinemic individuals were associated with relative insensitivity to the angiogenic effects of leptin [104]. Leptin induced EPCs and NO production has been shown to play critical roles in melanoma tumour growth induction [105]. Extending these findings to tumour angiogenesis, recent studies have implicated intratumoral leptin to exert proangiogenic effects stimulating tube formation and proliferation of endothelial cells. More importantly, the authors have also demonstrated the therapeutic potential of a peptide ObR antagonist in inhibiting these proangiogenic effects of leptin* via* the VEGF pathway [106]. Interestingly, leptin induced proliferation/migration as well as expression of proangiogenic molecules in breast cancer has been recently demonstrated to involve extensive crosstalk between Notch and interleukin-1 (NILCO) pathways [107].

### 4.8. Leptin and Vascular Inflammation

Leptin has been shown to upregulate various mediators of vascular inflammation like TNF-*α*, IL-2, IL-6, MCP-1, ROS, Th1-type cytokines, and TGF-*β* from endothelial cells and PBMCs [56, 108–110].* In vitro* studies have demonstrated leptin induced increases in tissue factor (TF) and cellular adhesion molecules (CAMs) expression in human coronary endothelial cells (HCAECs) via NF-*κβ* leading to increased procoagulant activity and leukocyte adhesion [111]. Additional molecules pivotal in vascular inflammation including PAI-1 (plasminogen activator inhibitor-1) and P-selectin have been documented to be induced upon leptin treatment [112, 113]. Clinical studies have shown a positive correlation with PAI-1, vWf, tPA, and plasma fibrinogen levels and an inverse relationship with protein C and tissue factor pathway inhibitor. These findings clearly demonstrate a strong link with circulating leptin and increased platelet activity observed in the metabolic syndrome [114–117]. It is therefore not surprising to note the decreased incidence of atherosclerosis in hyperlipidaemic mice (*ob/ob*; apoE^−/−^) [118].

### 4.9. Leptin-Adiponectin Ratio and Interactions

As discussed previously, converse actions of leptin and adiponectin in the vascular system have been widely studied. In obesity and diabetic metabolic abnormalities, coexistence of hypoadiponectinemia and hyperleptinemia is observed. Thereby, the leptin to adiponectin ratio (L : A) is higher in these subjects. Various clinical studies have been conducted to elucidate the relationship between L : A ratio and markers of atherosclerotic disease including carotid intima media thickness (CIMT) and pulse wave velocity [119–121]. In yet another clinical study, L : A ratio has been demonstrated as a useful biomarker for the prevalence of metabolic syndrome, in comparison with either leptin or adiponectin levels on their own. Additionally, visceral fat mass and cardiorespiratory fitness levels have been documented to influence this ratio [122]. Subjects with eNOS polymorphisms with or without hyperinsulinemia have a higher L : A ratio and are more prone for cardiovascular events, suggesting a genetic link in the associated risk factors [123]. Labruna et al. have demonstrated that high serum L/A ratio is positively correlated with serum triglyceride levels, serving as surrogate markers of vascular inflammation in “at-risk” young severely obese individuals, which is independent of waist circumference (WC) and BMI [124]. Additionally, L : A ratio represented a powerful independent predictor of intima media thickness (IMT), correlating with anthropometric, metabolic, and clinical parameters. Moreover the correlation with this ratio was much stronger than when compared individually [120]. Furthermore, components of the metabolic syndrome were correlated positively with leptin/HMW adiponectin ratio, independent of parameters including age, smoking status, exercise, low-density lipoprotein (LDL) cholesterol, and BMI [125].

However, in contrary to the above mentioned findings, L : A ratio failed to establish any significant differences in disease parameters, in a study conducted in patients with severe coronary heart disease [126]. It is noteworthy to mention that these above mentioned studies do differ in patient characteristics and pathological parameters leading to opposing results.

## 5. Conclusions/Future Directions

Adipose tissue secreted factors or adipokines have been implicated in facilitating communication between adipose tissue and vasculature comprising the adipovascular axis. Both proinflammatory and anti-inflammatory activities of these secreted adipokines seem to be crucial in creating a homeostatic response which remains disturbed in states of adipose tissue expansion. In addition to alterations in the circulating levels, the local (i.e., tissue concentration) availability of the activated forms of these adipokines has a significant bearing in influencing vascular function. For example, it is important to consider the actions of locally available gAD fragment of adiponectin, which could potentially drive leptin-induced effects. In-depth understanding of the mechanisms and properties of adipokine-receptor interactions and downstream signalling cascades may help in a clearer understanding of the pathogenesis of obesity-linked disorders. Studies investigating the vascular effects of various multimeric/cleaved forms of adipokines will help in developing novel therapeutic strategies and targets in counteracting obesity-related metabolic and CVDs. Additionally large multicentric clinical studies with strict inclusion-exclusion metabolic criteria need to be performed.

## Figures and Tables

**Table 1:** Differential effects of fAD, gAD, and leptin in endothelial cells.

Induced effect in endothelial cells [EC]	fAD (dose and time duration of response)	gAD (dose and time duration of response)	Leptin (dose and time duration of response)
Receptors	AdipoR1 and AdipoR2	Predominantly AdipoR1	OB-R (both short and long forms)

*In vitro *proliferation, migration, and angiogenesis	HUVECs-30 ug/mL-24 hrs-angiogenesis [31] HAECs-30 *μ*g/mL-24 hrs-↓migration [32] PAEs-1 *μ*g/mL-24 hrs-↓proliferation and migration [33] HAECs-0.5 *µ*g/mL-24 hrs-↓VEGF migration [34] HMECs-3.0 *µ*g/mL-24 hrs-↓proliferation [35]	BAECs-5 mg/mL-↓ox-LDL induced EC proliferation [36] EPCs-20 *μ*g/mL-24 hrs-migration-angiogenesis [37] HAECs-0.5 *µ*g/mL 24 hrs- ↓VEGF induced EC migration [34] HMECs-3.0 *µ*g/mL-24 hrs-proliferation, migration, and angiogenesis [35]	[(HUVECs)-(10–40 ng/mL)-24 hrs]-proliferation and angiogenesis [38]

EC inflammation and mediators	[(HAECs)-50 ug/mL-18 hrs-↓TNF-*α* induced NF-*κβ* [32]]	[HUVECs-10 ug/mL-8 hrs]- NF-*κβ* activation [39] [(HUVECs)-3 ug/mL-5 hrs]-↑procoagulability [40] [(HAECs)-10 ug/mL-8 hrs]-↑adhesion, VCAM-1/COX-2 *via* NF-*κβ* pathways [41]	[(HUVECs)-10-ng/mL-1 hr] NF-*κβ* activation, MCP-1 production, and ↑ROS production [42] [(HCAECs)-10 ng/mL-↑TF expression and activity, ↑VCAM-1, ICAM-1, and E-selectin expression and EC-monocyte adhesion [43]]

eNOS and NO production	[(HUVECs)-30 ug/mL-]-AMPK-eNOS phosphorylation [31]	[(EPCs)-5 *μ*g/mL-eNOS phosphorylation and NO production [37]]	[(HAECs)-10 ng/mL-eNOS phosphorylation and ↑NO production [44]].

The *in vitro* effects of fAD, gAD, and leptin differ on the concentration, time duration of peptide exposure, and the type of endothelial cells. BAECs: bovine aortic endothelial cells, EC: endothelial cell, eNOS: endothelial nitric oxide synthase, EPCs: endothelial progenitor cells, E-selectin: endothelial selectin, fAD: full length adiponectin, gAD: globular adiponectin, HAECs: human aortic endothelial cells, HCAECs: human coronary artery endothelial cells, HMECs: human microvascular endothelial cells, HUVECs: human umbilical vein endothelial cells, ICAM-1: intercellular cell adhesion molecule, MCP-1: monocyte chemoattractant protein-1, PAEs: porcine aortic endothelial cells, ROS: reactive oxygen species, TF: tissue factor, TNF*α*: tumour necrosis factor alpha, VCAM-1: vascular cell adhesion molecule, NF-*κβ*: nuclear factor kappa beta, and VEGF: vascular endothelial growth factor.

## Abbreviations

AdipoR1: Adiponectin receptor 1
AdipoR2: Adiponectin receptor 2
AMPK: AMP-activated protein kinase
cAMP: Cyclic adenosine monophosphate
eNOS: Endothelial nitric oxide synthase
fAD: Full length adiponectin
FGF-2: Fibroblast growth factor-2
gAD: Globular adiponectin
iNOS: Inducible nitric oxide synthase
IL-2/-6: Interleukin-2/-6
MCP-1: Monocyte chemoattractant protein-1
MMP-2/-9: Matrix metalloproteinase 2/9
NF-*κβ*: Nuclear factor *κβ*
NO: Nitric oxide
PI3K: Phosphatidylinositol 3-kinase
PKA: Protein kinase A
ROS: Reactive oxygen species
STAT3: Signal transducer and activator of transcription 3
TGF-*β*: Tumour growth factor beta
TNF-*α*: Tumour necrosis factor alpha
VCAM-1: Vascular cell adhesion molecule
VEGF: Vascular endothelial growth factor.
